# Association between alanine aminotransferase to high-density lipoprotein cholesterol ratio and nonalcoholic fatty liver disease: a retrospective cohort study in lean Chinese individuals

**DOI:** 10.1038/s41598-024-56555-8

**Published:** 2024-03-13

**Authors:** Changchun Cao, Zihe Mo, Yong Han, Jiao Luo, Haofei Hu, Dehua Yang, Yongcheng He

**Affiliations:** 1 Department of Rehabilitation, Shenzhen Dapeng New District Nan’ao People’s Hospital, No. 6, Renmin Road, Dapeng New District, Shenzhen, 518000 Guangdong China; 2 Department of Physical Examination, DongGuan Tungwah Hospital, Dongguan, China; 3 Department of Emergency, Shenzhen Second People’s Hospital, The First Affiliated Hospital of Shenzhen University, Shenzhen, 518000 Guangdong China; 4 Department of Nephrology, Shenzhen Second People’s Hospital, The First Affiliated Hospital of Shenzhen University, Shenzhen, 518000 Guangdong China; 5 Department of Pediatrics, Shenzhen Hengsheng Hospital, No. 20 Yintian Road, Xixiang Street, Baoan District, Shenzhen, 518000 Guangdong China; 6 Department of Nephrology, Affiliated Hospital of North Sichuan Medical College, No. 1 Maoyuan South Road, Nanchong, 637000 Sichuan China; 7 Department of Nephrology, Guangdong Province, Shenzhen Hengsheng Hospital, No. 20 Yintian Road, Xixiang Street, Baoan District, Shenzhen, 518000 China

**Keywords:** Alanine aminotransferase to high-density lipoprotein cholesterol ratio, Alanine aminotransferase, High-density lipoprotein cholesterol, NAFLD, Non-linear, Diseases, Endocrine system and metabolic diseases

## Abstract

There is limited research on the association between the alanine aminotransferase to high-density lipoprotein cholesterol ratio (ALT/HDL-C) ratio and nonalcoholic fatty liver disease (NAFLD). The purpose of the current research was to look into the connection between the ALT/HDL-C ratio and the risk of NAFLD in lean Chinese individuals. Between January 2010 and December 2014, 11,975 non-obese people participated in this prospective cohort research. The relationship between the ALT/HDL-C ratio and the risk of developing NAFLD was assessed using the Cox proportional-hazards regression model, Cox proportional hazards regression with cubic spline functions and smooth curve fitting, sensitivity analysis, and subgroup analyses. The ALT/HDL-C ratio’s potential value as a NAFLD prognostic marker was to be evaluated using the receiver operating characteristic curve analysis. A total of 5419 (45.253%) women comprised the research's participant population, and the research participants’ average age was 43.278 ± 14.941 years. The ALT/HDL-C ratio was 11.607 (7.973–17.422) at the median (interquartile ranges). 2087 (17.428%) patients had NAFLD diagnoses throughout a median follow-up of 24.967 months. The study's findings demonstrated a positive connection between the ALT/AHDL-C ratio and the incident NAFLD (HR = 1.037, 95% CI: 1.031–1.042) when adjusting for relevant factors. The ALT/HDL-C ratio and NAFLD risk had a nonlinear connection, with 12.963 as the ratio's inflection point. Effect sizes (HR) were 1.023 (95% CI: 1.017–1.029) and 1.204 (95% CI: 1.171–1.237), respectively, on the right and left sides of the inflection point. The sensitivity analysis also showed how reliable our findings were. According to subgroup analysis, those with BMI < 24 kg/m^2^ and DBP < 90 mmHg had a stronger correlation between the ALT/HDL-C ratio and NAFLD risk. The current study shows a positive and non-linear connection between the ALT/HDL-C ratio and NAFLD risk in lean Chinese individuals. When the ALT/HDL-C ratio is less than 12.963, it is significantly linked to NAFLD. Therefore, from a therapy standpoint, it is advised to keep the ALT/HDL-C ratio less than the inflection point.

## Introduction

The world’s most common chronic liver disease is a non-alcoholic fatty liver disease (NAFLD), characterized by fat buildup inside hepatocytes (hepatic steatosis). Hepatocellular carcinoma incidence strongly correlates with its clinical symptoms, including hepatic steatosis, steatohepatitis, fibrosis, and cirrhosis^1–3^. Although NAFLD has also frequently been seen in obese people^4^, the percentage of NAFLD in lean or non-obese patients is increasing^5–7^. Within the United States, the prevalence of NAFLD is estimated at 23%^8^, with up to 7–20% of lean individuals suffering from hepatic steatosis^9,10^. Hepatic steatosis has been found in 8–19% of lean people in Asian populations^11^. Additionally, patients who are lean and have NAFLD are more prone to develop metabolic syndrome and other severe conditions, such as type 2 diabetes mellitus (T2DM), severe cardiovascular disease, and liver disease^12–15^. Furthermore, numerous investigations have established a link between low-density lipoprotein cholesterol (LDL-C) and NAFLD^16,17^. Concurrently, recent research indicated that even LDL-C levels considered within the normal range could significantly impact NAFLD's occurrence and widespread nature^18^. Therefore, it can still be important to identify lean individuals with a normal range of LDL-C who are at risk for NAFLD.

Alanine aminotransferase (ALT), a commonly used marker to assess hepatocyte damage, is a well-established indicator of non-alcoholic fatty liver disease (NAFLD). It has a strong connection to the prevalence of NAFLD^19,20^. Elevated ALT levels are also linked to NAFLD with a more severe histological spectrum, including fibrosis and non-alcoholic steatohepatitis (NASH), according to research^21,22^. Conversely, decreased levels of high-density lipoprotein cholesterol (HDL-C) are a risk factor for the development of metabolic syndrome^23,24^. NAFLD and the components of metabolic syndrome are connected. It has been proposed that NAFLD is sometimes viewed as the metabolic syndrome's hepatic manifestation^25^. According to several recent research, the risk of T2DM and NAFLD was correlated with HDL-C and ALT levels^20,26^. Recent cohort research also found a positive correlation between higher alanine aminotransferase to high-density lipoprotein cholesterol (ALT/HDL-C) ratio levels and T2DM^27^. However, there was no proof that the ALT/HDL-C ratio increased the incident NFLAD. We conducted cohort research to explore this theory and learn how the ALT/HDL-C ratio affects incident NAFLD in lean Chinese individuals with a normal range of LDL-C.

## Methods

### Data source

From January 2010 to December 2014, participants at Wenzhou Medical Center, Wenzhou People's Hospital, China, were enrolled in this retrospective cohort study^28^. DATADRYAD database made the initial data provided by Sun et al.^28^ accessible for free download.

### Study participants

In order to minimize selection bias, the individuals were obtained consecutively from Wenzhou People’s Hospital in China. Their identity information was encoded into an untraceable code to maintain the participants’ anonymity. Every procedure involving patients at Wenzhou People's Hospital was approved by the clinical research ethics committee in line with the Helsinki Declaration^28^. In addition, this study has been approved by the Ethics Committee of the Shenzhen Dapeng New District Nan’ao People’s Hospital (2022082201).

**Figure 1:**
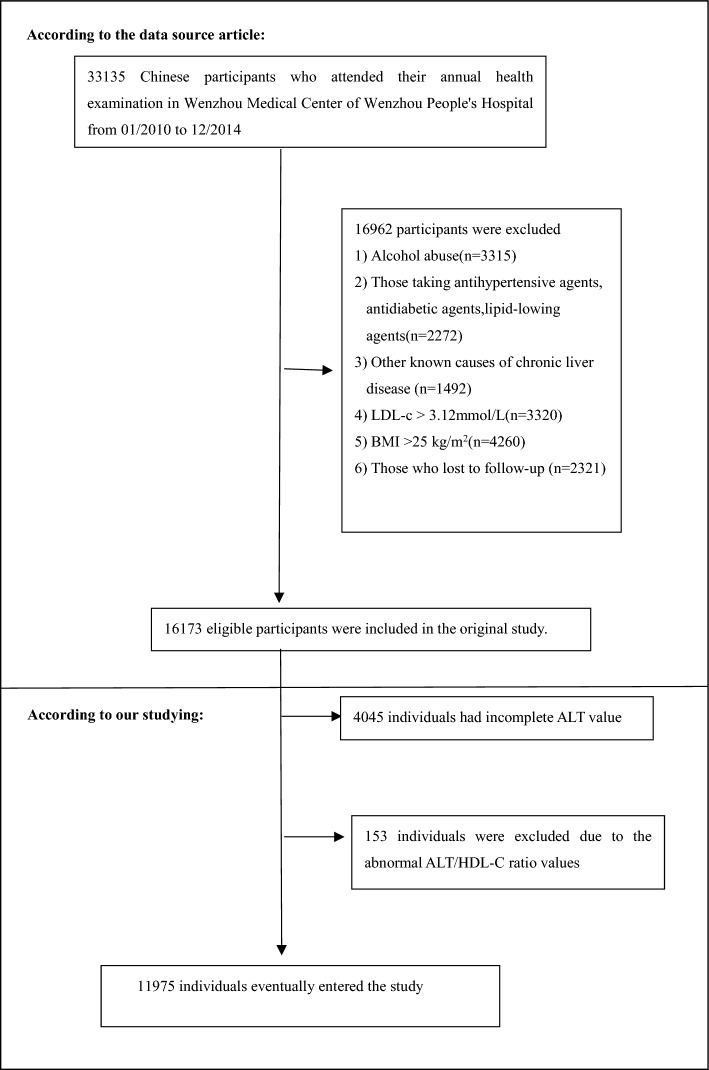
Study population.

Between January 2010 and December 2014, 33,153 Chinese people without NAFLD in the original research who underwent a health evaluation met the inclusion criteria for the initial investigation^28^. 21,178 subjects were eliminated, leaving 11,975 subjects for data analysis in the current research. There were some exclusion standards: (1) LDL-C > 3.12 mmol/L; (2) body mass index (BMI) ≥ 25 kg/m^2^; (3) taking lipid-lowering, anti-diabetic agents or anti-hypertensive; (4) excessive drinking (not less than 140 g per week for men or 70 g per week for women); (5) known chronic liver diseases, such as autoimmune hepatitis, NAFLD, or viral hepatitis; (6) missing data or lost to follow-up. In addition, individuals with missing ALT values (n = 4045) and individuals with abnormal ALT/HDL-C ratio values (three standard deviations above or below the mean) (n = 153) were not included in the current investigation (Fig. 1).

### ALT/HDL-C ratio

HDL-C and ALT levels were assessed in fasting blood samples using the Abbott AxSYM automatic biochemical analyzer. The Abbott Abbott AxSYM automatic biochemical analyzer used an enzymatic assay to measure ALT levels and a direct selective immunoinhibition assay to measure HDL-C. Then, by dividing ALT by HDL-C, we created a new variable that served as the foundation for our study.

### Diagnosis of incident NAFLD

According to the Chinese Liver Disease Association's recommendations^29^, the existence of NAFLD was determined by ultrasonography based on the following criteria: (1) diffusely boosted near-field echoes and progressively attenuated far-field echoes in the liver region; (2) unclear display of intrahepatic structure; (3) enlarged liver with a round and blunt border; (4) Weakened hepatic blood flow signal; (5) right liver lobe and diaphragm being unclear or incomplete.

### Covariates

The factors for this investigation were selected considering prior research and our clinical expertise. Personal information, such as weight, gender, height, and age, was collected based on a standardized spreadsheet. The subjects were seated in a peaceful area, and their diastolic blood pressure (DBP) and systolic pressure (SBP) were measured using an automated blood pressure monitor. BMI was computed as the weight (kg) ratio and the square of height (m^2^). The following laboratory parameters were measured in venous blood after an overnight fast: triglyceride (TG), creatinine (Cr), γ-glutamyl transpeptidase (GGT), LDL-C, aspartate aminotransferase (AST), uric acid (UA), fasting plasma glucose (FPG), total cholesterol (TC), and alkaline phosphatase (ALP).

### Missing data processing

There were 15 (0.125%), 15 (0.125%), and 2 (0.017%) subjects with missing data for SBP, DBP, and GGT, respectively. The missing data were handled by applying multiple imputations. Age, gender, AST, BMI, ALP, SBP, GGT, TC, DBP, UA, TG, Cr, FPG, and LDL-C were all added to the imputation model. Missing-at-random assumptions were employed in missing data analysis processes^30,31^.

### Statistical analysis

Statistical analysis was employed using Empower Stats (version 4.1) and R software version 3.4.3.

Using the quartiles of the ALT/HDL-C, the current research separated the subjects into four groups. The median (interquartile ranges) or mean ± standard deviation (SD) were shown for continuous data, while frequencies and percentages were shown for categorical data. The present research utilized χ^2^ (categorical data), the Kruskal–Wallis H test (skewed distribution), and the one-way ANOVA test (normal distribution) to examine differences between ALT/HDL-C ratio groups.

The current research utilized univariate and multivariate Cox proportional-hazards regression models to test the correlation between ALT/HDL-C ratio and NAFLD, including Model 1 (no adjustment for covariates), Model 2 (adjusted DBP, gender, age, SBP, and BMI) and Model 3 (adjusted AST, ALP, GGT, UA, Cr, FPG, TG, TC, LDL-C plus the same parameters as model 2). The models generated effect sizes (HR) and 95% confidence intervals (CI), which were adjusted for covariates that resulted in a 10% or greater change in HR^32^. Moreover, no covariates were excluded from the Cox proportional-hazards regression models according to the screening results for collinearity.

The current research carried out several sensitivity analyses to evaluate the robustness of our findings. In order to scrutinize the findings of the ALT/HDL-C ratio as a continuous variable and investigate the likelihood of non-linearity, the current research ascertained the P-value for the trend by categorizing the ALT/HDL-C ratio into quartiles. TG^33^ and obesity^4^ are strongly linked to NAFLD. We thus excluded patients with TG ≥ 1.7 mmol/L and BMI ≥ 24 kg/m^2^ from further sensitivity analyses looking at the correlation between the ALT/HDL-C ratio and NAFLD risk. Additionally, the current research utilized a generalized additive model (GAM) to further integrate the continuous covariate as a curve in the equation to validate the findings (model 4).

To accommodate the non-linear correlation between the ALT/HDL-C ratio and NAFLD, the current research employed a Cox proportional hazards regression model with cubic spline functions and smooth curve fitting^34^. The two-piecewise Cox proportional-hazards regression model was utilized to shed light on the inflection point and threshold effect of the ALT/HDL-C ratio and NAFLD.

The current research used a stratified Cox proportional-hazards regression model to analyze multiple subgroups (DBP, gender, age, SBP, and BMI). First, according to the clinical cutpoints, continuous variables for age (< 60, ≥ 60 years), DBP (< 90, ≥ 90 mmHg), BMI (< 24, ≥ 24 kg/m^2^), and SBP (< 140, ≥ 140 mmHg) were transformed into categorical variables. Second, we adjusted each stratification for all variables (DBP, gender, age, SBP, BMI, TC, AST, UA, GGT, TG, ALP, Cr, FPG, LDL-C) except the stratification factor itself. The likelihood ratio test was utilized in models with and without interaction terms to determine the presence of such terms^35^.

The Receiver Operating Characteristic (ROC) curve was employed to evaluate the predictive capacity of HDL-C, ALT, and ALT/HDL-C ratio in relation to NAFLD risk. The STROBE criteria were followed while writing up all of the results. Two-tailed test to determine statistical significance at P less than 0.05.

### Ethics approval and consent to participate

The study was conducted following the Declaration of Helsinki and was approved by the Clinical Research Ethics Committee in Wenzhou People’s Hospital. Informed consent was obtained from all subjects and/or their legal guardian(s). In addition, the study has also been approved by the Ethics Committee of the Shenzhen Dapeng New District Nan'ao People's Hospital (2022082201).

## Results

### Characteristics of subjects

In the present study, 11,975 subjects deemed free of NAFLD at baseline were included. The mean age of the current research was 43.278 ± 14.941 years, with 5419 (45.253%) of the subjects being female. The average age was 43.278 ± 14.941 years, and 5419 (45.253%) were female. 2087 (17.428%) patients had NAFLD diagnoses throughout a median follow-up of 24.967 months. Table 1 provides the fundamental indicators, laboratory tests, and other factors of participants included in the study. Using the quartiles of the ALT/HDL-C ratio (Q1 ≤ 7.971; 7.971 < Q2 ≤ 11.602; 11.602 < Q3 ≤ 17.419; Q4 > 17.419). The other three groups had higher ALP, DBP, ALT, FPG, Cr, BMI, GGT, UA, TG, AST, SBP, and lower HDL-C, TC compared to the Q1 group (ALT/HDL-C ratio ≤ 7.971). Additionally, the Q4 group (ALT/HDL-C ratio > 17.419) had a larger percentage of men.

**Table 1:** The baseline characteristics of participants.

ALT/HDL-C ratio	Q1 (≤ 7.971)	Q2 (7.971–11.602)	Q3 (11.602–17.419)	Q4 (> 17.419)	P-value
Participants	2994	2992	2995	2994	
Gender					< 0.001
Female	1485 (49.599%)	1366 (45.655%)	1311 (43.773%)	1257 (41.984%)	
Male	1509 (50.401%)	1626 (54.345%)	1684 (56.227%)	1737 (58.016%)	
Age (years)	43.048 ± 15.103	43.308 ± 15.080	43.803 ± 15.126	42.955 ± 14.439	0.121
SBP (mmHg)	117.895 ± 17.484	121.505 ± 17.128	124.473 ± 16.762	125.592 ± 15.250	< 0.001
DBP (mmHg)	70.888 ± 10.001	72.938 ± 10.181	75.030 ± 10.468	76.221 ± 10.057	< 0.001
BMI (kg/m^2^)	20.558 ± 1.992	21.294 ± 1.987	21.947 ± 1.888	22.597 ± 1.693	< 0.001
ALT (IU/L)	10.638 ± 2.606	14.599 ± 3.096	19.147 ± 4.362	31.681 ± 12.150	< 0.001
AST (IU/L)	18.656 ± 3.769	20.650 ± 4.087	22.798 ± 5.070	27.995 ± 9.244	< 0.001
GGT (IU/L)	17 (14, 21)	19 (15, 25)	24 (19, 32)	32 (23, 49)	< 0.001
ALP (IU/L)	64.070 ± 18.515	70.090 ± 23.773	74.476 ± 21.831	79.954 ± 24.511	< 0.001
HDL-C (mmol/L)	1.732 ± 0.332	1.512 ± 0.296	1.352 ± 0.286	1.209 ± 0.279	< 0.001
TG (mmol/L)	0.90 (0.71, 1.140)	1.05 (0.81, 1.38)	1.25 (0.95, 1.69)	1.55 (1.13, 2.23)	< 0.001
TC (mmol/L)	4.671 ± 0.725	4.599 ± 0.720	4.566 ± 0.729	4.593 ± 0.759	< 0.001
LDL-C (mmol/L)	2.202 ± 0.466	2.269 ± 0.473	2.295 ± 0.463	2.324 ± 0.473	< 0.001
FPG (mmol/L)	5.063 ± 0.625	5.182 ± 0.737	5.266 ± 0.874	5.332 ± 0.997	< 0.001
UA (μmol/L)	254.902 ± 85.821	279.791 ± 87.351	306.818 ± 84.356	330.421 ± 81.953	< 0.001
Cr (μmol/L)	77.433 ± 23.385	81.962 ± 24.191	86.806 ± 30.280	87.599 ± 20.240	< 0.001
ALT/HDL-C ratio	6.196 ± 1.227	9.682 ± 1.043	14.218 ± 1.694	26.426 ± 8.426	< 0.001

Values are n (%) or mean ± SD or median (quartile).

*ALT/HDL-C ratio* alanine aminotransferase to high-density lipoprotein cholesterol ratio, *BMI* body mass index, *SBP* systolic blood pressure, *DBP* diastolic blood pressure, *ALT* alanine aminotransferase, *AST* aspartate aminotransferase, *GGT* gamma-glutamyl transferase, *ALP* alkaline phosphatase, *HDL-C* high-density lipoprotein cholesterol, *TC* total cholesterol, *TG* triglycerides, *FPG* fasting plasma glucose, *UA* uric acid, *Cr* creatinine.

### The incidence rate of NAFLD

2087 (17.428%) patients had NAFLD diagnoses throughout a median follow-up of 24.967 months. All persons’ cumulative incidence rate and each ALT/HDL-C ratio group’s cumulative incidence rate were 7.128, 1.659, 3.932, 8.187, and 14.734 per 100 person-years, respectively (Table 2). All persons’ incidence rates and each ALT/HDL-C ratio group’s incidence rate were 17.428% (16.748–18.108%), 4.008% (3.305–4.711%), 9.659% (8.600–10.718%), 20.334% (18.892–21.776%), and 35.705% (33.988–37.422%), respectively (Table 2). In contrast to the group with the lowest ALT/HDL-C ratio, participants with greater ALT/HDL-C ratios showed higher incidence rates of NAFLD (P < 0.001 for trend).

**Table 2:** Incidence rate of incident NAFLD.

ALT/HDL-C ratio	Participants (n)	NAFLD events (n)	Incidence rate (95% CI) (%)	Per 100 person-year
Total	11,975	2087	17.428 (16.748–18.108)	7.128
Q1	2994	120	4.008 (3.305–4.711)	1.659
Q2	2992	289	9.659 (8.600–10.718)	3.932
Q3	2995	609	20.334 (18.892–21.776)	8.187
Q4	2994	1069	35.705 (33.988–37.422)	14.734
P for trend			< 0.001	< 0.001

*ALT/HDL-C ratio* alanine aminotransferase to high-density lipoprotein cholesterol ratio, *CI* confidence interval, *NAFLD* nonalcoholic fatty liver disease.

**Figure 2:**
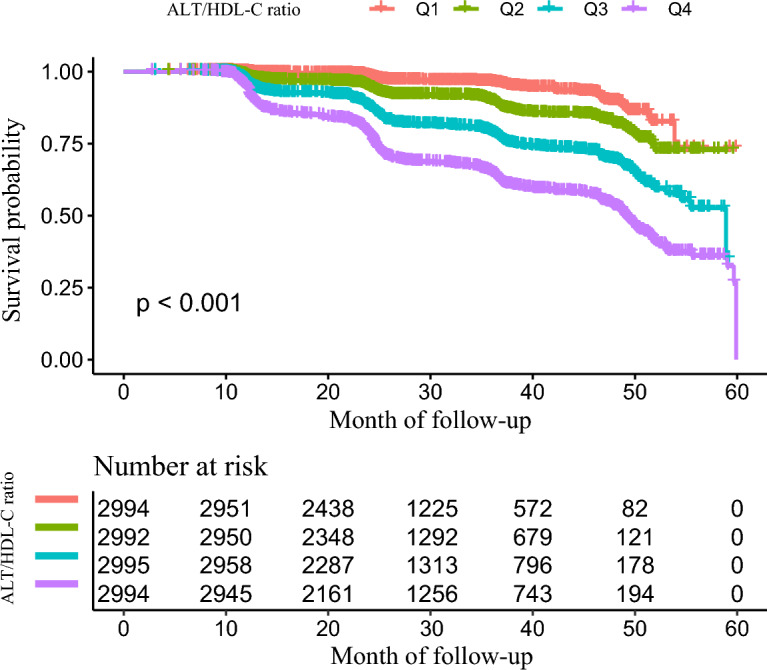
Kaplan–Meier event-free survival curve. Kaplan–Meier event-free survival curve. Kaplan–Meier analysis of incident NAFLD based on the ALT/HDL-C ratio quartiles (log-rank, P < 0.001).

Figure 2 displays the Kaplan–Meier survival curves for NAFLD-free survival probability divided by the ALT/HDL-C ratio group. The groups with different ALT/HDL-C ratios had significantly different chances of NAFLD-free survival (log-rank test, P < 0.001). This current study indicated that the Q4 group (ALT/HDL-C ratio > 17.419) had the highest risk of developing NAFLD, and the chance of NAFLD-free survival g steadily decreased as the ALT/HDL-C ratio increased.

### The correlation between ALT/HDL-C ratio and NAFLD

The relevant data were undergoing a univariate analysis, which revealed that DBP, age, SBP, BMI, TC, AST, UA, GGT, TG, ALP, Cr, FPG, LDL-C, and ALT/HDL-C ratio were positively correlated with NAFLD in Table 3.

**Table 3:** The results of the univariate Cox proportional hazards model.

	Statistics	HR (95% CI)	*P* value
Gender			0.29041
Female	5419 (45.253%)	Ref	
Male	6556 (54.747%)	1.048 (0.961, 1.142)	
Age (years)	43.278 ± 14.941	1.006 (1.003, 1.009)	< 0.001
SBP (mmHg)	122.366 ± 16.940	1.014 (1.012, 1.017)	< 0.001
DBP (mmHg)	73.770 ± 10.379	1.034 (1.030, 1.038)	< 0.001
BMI (kg/m^2^)	21.599 ± 2.040	1.671 (1.622, 1.721)	< 0.001
AST (IU/L)	22.525 ± 6.902	1.020 (1.016, 1.025)	< 0.001
GGT (IU/L)	29.453 ± 29.618	1.006 (1.005, 1.007)	< 0.001
ALP (IU/L)	72.148 ± 23.025	1.008 (1.007, 1.009)	< 0.001
TG (mmol/L)	1.368 ± 0.900	1.296 (1.275, 1.318)	< 0.001
TC (mmol/L)	4.607 ± 0.735	1.332 (1.257, 1.411)	< 0.001
LDL-C (mmol/L)	2.272 ± 0.471	1.751 (1.587, 1.931)	< 0.001
FPG (mmol/L)	5.211 ± 0.826	1.227 (1.195, 1.260)	< 0.001
UA (μmol/L)	292.986 ± 89.496	1.003 (1.002, 1.003)	< 0.001
Cr (μmol/L)	83.451 ± 25.125	1.002 (1.001, 1.003)	< 0.001
ALT/HDL-C ratio	14.131 ± 8.809	1.052 (1.048, 1.055)	< 0.001

Values are n (%) or mean ± SD.

*ALT/HDL-C ratio* alanine aminotransferase to high-density lipoprotein cholesterol ratio, *BMI* body mass index, *SBP* systolic blood pressure, *DBP* diastolic blood pressure, *AST* aspartate aminotransferase, *GGT* gamma-glutamyl transferase, *ALP* alkaline phosphatase, *TC* total cholesterol, *TG* triglycerides, *FPG* fasting plasma glucose, *UA* uric acid, *Cr* creatinine.

The ALT/HDL-C ratio and NAFLD risk were shown to be related by employing the multivariate Cox proportional-hazards regression model (Table 4). A 5.2% increase in the risk of NAFLD was connected to a rise of 1 unit in the ALT/HDL-C ratio in Model 1 (HR = 1.052, 95% CI: 1.048–1.055, P < 0.001). When we solely made demographic adjustments (DBP, gender, age, SBP, and BMI) in Model 2, the risk of NAFLD rose by 3.4% for every extra unit of ALT/HDL-C ratio (HR = 1.034, 95% CI: 1.030–1.038, P < 0.001). In Model 3, the incident NAFLD increased by 3.7% for every extra unit of the ALT/HDL-C ratio when adjusting for relevant factors (DBP, gender, age, SBP, BMI, TC, AST, UA, GGT, TG, ALP, Cr, FPG, and LDL-C) (HR = 1.037, 95% CI: 1.031–1.042, P < 0.001). Additionally, the Q4 group (ALT/HDL-C ratio > 17.419) was related to an elevated risk for NAFLD when we used the lowest quartile as a reference (Model 1, Q4: HR = 8.440, 95% CI: 6.987, 10.195, P < 0.001) (Model 2, Q4: HR = 4.469, 95% CI: 3.688–5.416, P < 0.001) (Model 3, Q4: HR = 4.417, 95% CI: 3.575–5.457, P < 0.001) (Table 4).

**Table 4:** Relationship between ALT/HDL-C ratio and the incident NAFLD in different models.

Variable	Model 1 (HR, 95% CI, P)	Model 2 (HR, 95% CI, P)	Model 3 (HR, 95% CI, P)	Model 4 (HR, 95% CI, P)
ALT/HDL-C ratio	1.052 (1.048, 1.055) < 0.001	1.034 (1.030, 1.038) < 0.001	1.037 (1.031, 1.042) < 0.001	1.028 (1.022, 1.034) < 0.00001
ALT/HDL-C ratio (quartile)				
Q1	Ref	Ref	Ref	Ref
Q2	2.317 (1.873, 2.867) < 0.001	1.796 (1.451, 2.223) < 0.001	1.795 (1.447, 2.226) < 0.001	1.580 (1.270, 1.965) < 0.001
Q3	4.727 (3.886, 5.750) < 0.001	3.011 (2.472, 3.668) < 0.001	2.951 (2.408, 3.618) < 0.001	2.350 (1.899, 2.909) < 0.001
Q4	8.440 (6.987, 10.195) < 0.001	4.469 (3.688, 5.416) < 0.001	4.417 (3.575, 5.457) < 0.001	3.317 (2.642, 4.164) < 0.001
P for trend	< 0.001	< 0.001	< 0.001	< 0.001

Model 1: we did not adjust for other covariants.

Model 2: we adjusted for DBP, gender, age, SBP, and BMI.

Model 3: we adjusted for DBP, gender, age, SBP, BMI, TC, AST, UA, GGT, TG, ALP, Cr, FPG, and LDL-C.

Model 4: we adjusted for DBP, gender, age, SBP, BMI, TC, AST, UA, GGT, TG, ALP, Cr, FPG, and LDL-C. However, continuous covariates were adjusted as nonlinearity.

*HR* hazard ratio, *CI* confidence interval, *Ref* reference, *ALT/HDL-C ratio* alanine aminotransferase to high-density lipoprotein cholesterol ratio, *NAFLD* nonalcoholic fatty liver disease.

### Sensitive analysis

The continuity covariate was added into the equation as a curve in the current research employing a GAM in Model 4 (HR = 1.028, 95% CI: 1.022–1.034, P < 0.001) (Table 4). The ALT/HDL-C ratio remained strongly linked with NAFLD even after the present investigation excluded patients with BMI ≥ 24 kg/m^2^ and TG > 1.7 mmol/L for sensitivity analysis in Table 5. The outcomes of each sensitivity analysis demonstrated the robustness of the association between the ALT/HDL-C ratio and the NAFLD.

**Table 5:** Relationship between the ALT/HDL-C ratio and incident NAFLD in different sensitivity analyses.

Exposure	Model 5 (HR, 95% CI, P)	Model 6 (HR, 95% CI, P)
ALT/HDL-C ratio	1.043 (1.036, 1.049) < 0.001	1.031 (1.022, 1.039) < 0.001
ALT/HDL-C ratio (quartile)		
Q1	Ref	Ref
Q2	2.035 (1.575, 2.629) < 0.001	1.577 (1.234, 2.016) < 0.001
Q3	3.221 (2.522, 4.114) < 0.001	2.269 (1.786, 2.882) < 0.001
Q4	5.031 (3.908, 6.477) < 0.001	3.127 (2.407, 4.063) < 0.001
P for trend	< 0.001	< 0.001

Model 5 was sensitivity analysis after excluding those with BMI ≥ 24 kg/m^2^. We adjusted DBP, gender, age, SBP, BMI, TC, AST, UA, GGT, TG, ALP, Cr, FPG, and LDL-C.

Model 6 was sensitivity analysis after excluding those with TG ≥ 1.7 mmol/L. We adjusted DBP, gender, age, SBP, BMI, TC, AST, UA, GGT, TG, ALP, Cr, FPG, and LDL-C.

*HR* hazard ratios, *CI* confidence, *Ref* reference, *ALT/HDL-C ratio* alanine aminotransferase to high-density lipoprotein cholesterol ratio, *NAFLD* nonalcoholic fatty liver disease.

### The nonlinear correlation between the ALT/HDL-C ratio and NAFLD risk

**Figure 3:**
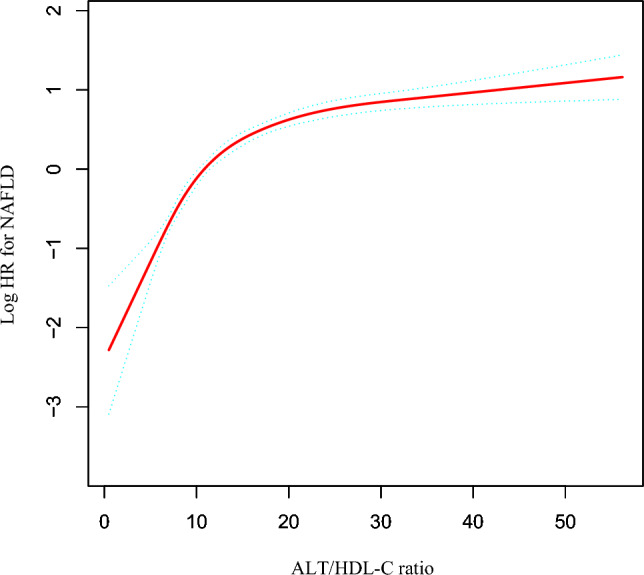
The nonlinear relationship between the ALT/HDL-C ratio and incident NAFLD. A nonlinear relationship was detected after adjusting for DBP, gender, age, SBP, BMI, TC, AST, UA, GGT, TG, ALP, Cr, FPG, and LDL-C.

The correlation between the ALT/HDL-C ratio and NAFLD was investigated employing the Cox proportional hazards regression model with cubic spline functions in Fig. 3. After controlling for DBP, gender, age, SBP, BMI, TC, AST, UA, GGT, TG, ALP, Cr, FPG, and LDL-C, a non-linear connection between the ALT/HDL-C ratio and NAFLD was revealed in Table 6 (log-likelihood ratio test P < 0.001). We first used a recursive method to identify the ALT/HDL-C ratio’s inflection point (12.963). Subsequently, a two-piecewise Cox proportional-hazards regression model was utilized to estimate the HR and 95% CI on either side of the inflection point. On the left side of the inflection point, the HR was 1.204 (95% CI: 1.171–1.237). On the right side of the inflection point, the HR was 1.023 (95% CI: 1.017–1.029).

**Table 6:** The result of the two-piecewise Cox regression model.

Incident NAFLD	HR (95% CI),	P
Fitting model by standard Cox regression	1.037 (1.031, 1.042)	< 0.001
Fitting model by two-piecewise Cox regression		
The inflection point of ALT/HDL-C ratio	12.963	
≤ 12.963	1.204 (1.171, 1.237)	< 0.001
> 12.963	1.023 (1.017, 1.029)	< 0.001
P for the log-likelihood ratio test	< 0.001	

We adjusted for DBP, gender, age, SBP, BMI, TC, AST, UA, GGT, TG, ALP, Cr, FPG, and LDL-C.

*HR* hazard ratios, *CI* confidence, *ALT/HDL-C ratio* alanine aminotransferase to high-density lipoprotein cholesterol ratio, *NAFLD* nonalcoholic fatty liver disease.

### The results of the subgroup analysis

There was no significant interaction between age, gender, and SBP in any predetermined or exploratory subgroups assessed (Table 7). Conversely, valuable interactions in BMI and DBP were found. More specifically, those with DBP < 90 mmHg (HR = 1.040, 95% CI: 1.034–1.045, P < 0.001) and BMI < 24 kg/m^2^ (HR = 1.057, 95% CI: 1.051–1.064, P < 0.001) had higher correlations between the ALT/HDL-C ratio and NAFLD. In comparison, individuals with DBP ≥ 90 mmHg (HR = 1.026, 95% CI: 1.015–1.037, P < 0.001) and BMI ≥ 24 kg/m^2^ (HR = 1.030, 95% CI: 1.021–1.039, P < 0.001) had lesser associations.

**Table 7:** Effect size of ALT/HDL-C ratio on NAFLD in prespecified and exploratory subgroups.

Characteristic	No of patients	Effect size (95% CI)	P value	P for interaction
Age (years)				0.484
< 60	10,125	1.037 (1.031, 1.043)	< 0.001	
≥ 60	1850	1.033 (1.020, 1.045)	< 0.001	
Gender				0.150
Female	5419	1.034 (1.027, 1.041)	< 0.001	
Male	6556	1.039 (1.033, 1.046)	< 0.001	
BMI (kg/m^2^)				< 0.001
< 24	10,382	1.057 (1.051, 1.064)	< 0.001	
≥ 24	1593	1.030 (1.021, 1.039)	< 0.001	
SBP (mmHg)				0.375
< 90	10,221	1.038 (1.032, 1.044)	< 0.001	
≥ 90	1754	1.033 (1.024, 1.043)	< 0.001	
DBP (mmHg)				0.012
< 140	10,981	1.040 (1.034, 1.045)	< 0.001	
≥ 140	994	1.026 (1.015, 1.037)	< 0.001	

Note 1: Above model adjusted for we adjusted for DBP, gender, age, SBP, BMI, TC, AST, UA, GGT, TG, ALP, Cr, FPG, and LDL-C.

Note 2: The model is not adjusted for the stratification variable in each case.

*HR* hazard ratios, *CI* confidence, *ALT/HDL-C ratio* alanine aminotransferase to high-density lipoprotein cholesterol ratio, *NAFLD* nonalcoholic fatty liver disease, *BMI* body mass index, *SBP* systolic blood pressure, *DBP* diastolic blood pressure.

### NAFLD prediction using the ALT/HDL-C ratio

**Figure 4:**
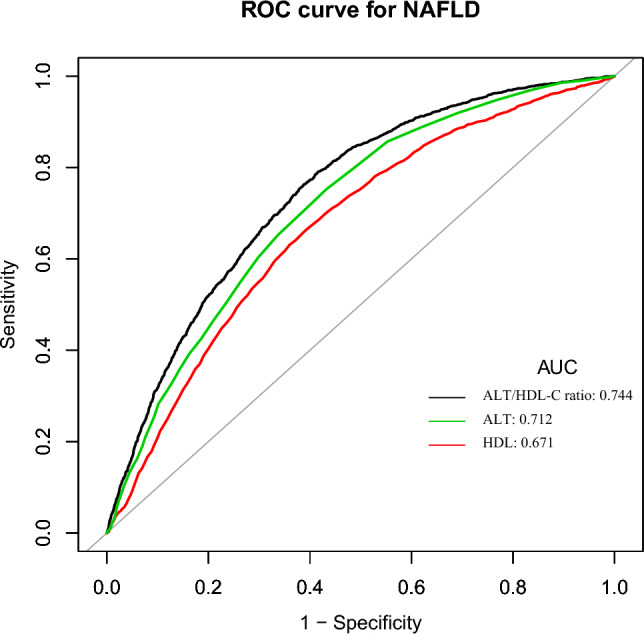
The ALT/HDL-C ratio for predicting NAFLD in all participants by ROC analyses.

The predictive capacity of HDL-C, ALT, and ALT/HDL-C ratio in relation to NAFLD risk was further evaluated using a ROC curve (Fig. 4). Areas under the curves are listed for each variable in the following table in Fig. 4: HDL-C: 0.671; ALT: 0.712; ALT/HDL-C ratio: 0.744. The best cut-off value was 1.365, 16.500, and 12.385, respectively, based on the Youden index of the ALT/HDL-C ratio, ALT, and HDL-C of 0.375, 0.320, and 0.271 (Table 8). The ALT/HDL-C ratio’s capacity to predict NAFLD risk was better than that of other variables, as evidenced by the fact that its Youden index and AUC were the biggest in Table 8.

**Table 8:** Areas under the receiver operating characteristic curves (AUROC) for each evaluated parameter in identifying NAFLD.

Test	AUROC	95% CI	Best threshold	Specificity	Sensitivity	Youden Index
ALT/HDL-C ratio	0.744	0.733–0.755	12.385	0.612	0.763	0.375
ALT	0.712	0.701–0.724	16.500	0.606	0.665	0.320
HDL-C	0.671	0.701–0.724	1.365	0.568	0.752	0.271

*CI* confidence, *ALT/HDL-C ratio* alanine aminotransferase to high-density lipoprotein cholesterol ratio, *NAFLD* nonalcoholic fatty liver disease, *ALT* alanine aminotransferase, *HDL-C* high-density lipoprotein cholesterol.

## Discussion

This retrospective investigation found that in lean Chinese individuals, a heightened ALT/HDL-C ratio was found to be associated with an increased risk of NAFLD. The inflection point of the nonlinear association between the ALT/HDL-C ratio and NAFLD was 12.963. Our findings revealed a statistically significant positive correlation between the ALT/HDL-C ratio level and NAFLD when it was 12.963 (HR = 1.204, 95% CI: 1.171–1.237). It was also discovered that BMI and DBP might change the link to the ALT/HDL-C ratio and NAFLD. In the population with DBP < 90 mmHg and BMI < 24 kg/m^2^, greatly stronger relationships were detected. According to the research mentioned above, the ALT/HDL-C ratio might serve as a benchmark for the main prevention of NAFLD in lean Chinese individuals ith a normal range of LDL-C.

A recent historical Japanese cohort study including 15,342 participants showed a positive correlation between elevated ALT/HDL-C ratio levels and T2DM (HR: 1.01, 95% CI: 1.00–1.02, P = 0.049)^27^. Based on a comprehensive literature search, it was determined that no current studies have examined the correlation between the ALT/HDL-C ratio and NAFLD risk. However, recent research has indicated a positive correlation between ALT levels and incident NAFLD^20,36^ and a negative correlation between HDL-C and the occurrence of NAFLD. In addition, a cross-sectional study that included 3311 participants found that ALT levels were an independent risk factor for NAFLD, even within the normal ALT range (OR = 4.98, 95% CI: 3.41–7.27)^20^. A multivariate Mendelian randomization analysis found that HDL-C was an independent protective factor for NAFLD (OR: 0.776, 95% CI: 0.604–0.998)^26^. Although there is no data on the association between the ALT/HDL-C ratio and the likelihood of developing NAFLD, an increase in the ratio indicates either an increase in ALT or a reduction in HDL-C. Consequently, our data support the aforementioned findings that ALT/HDL-C ratio levels are substantially correlated with NAFLD. What’s more, the sensitivity analysis showed that this link is still present in people with TG < 1.7 mmol/L or BMI < 24 kg/m^2^. The above results have demonstrated the consistency of the correlation between the ALT/HDL-C ratio and NALFD risk. This discovery offers a justification for mitigating the likelihood of NAFLD by decreasing the ALT/HDL-C ratio. Furthermore, we assessed the predictive capacity of the ALT/HDL-C ratio, HDL-C, and ALT for NAFLD using a ROC curve. We determined that the ALT/HDL-C ratio exhibited superior predictive ability compared to ALT or HDL-C in isolation. An elevated ALT/HDL-C ratio signifies an elevated risk of developing NAFLD. A higher ALT/HDL-C ratio during the follow-up period indicates an elevated risk of developing NAFLD, alerting individuals to modify their lifestyle choices to reduce the incidence of NAFLD proactively.

Uncertainty surrounds the correlation between an elevated ALT/HDL-C ratio and NAFLD. There are, however, two explanations that could fit this event. First, research has demonstrated that high ALT is linked to metabolic syndrome and insulin resistance^37,38^. NAFLD is significantly impacted by insulin resistance^39,40^. As a result, ALT may affect NAFLD development by influencing insulin resistance. Given the significant role of oxidative stress in the onset and advancement of non-alcoholic fatty liver disease^41^, it is implied that the potential involvement of HDL-C's antioxidant activity may be involved in the pathophysiology of non-alcoholic fatty liver disease^42^. A high ratio of alanine ALT to HDL-C levels, characterized by reduced HDL-C and elevated ALT, may indicate NAFLD.

The ALT/HDL-C ratio and incident NAFLD were shown to have a non-linear correlation using a two-piecewise Cox proportional hazards regression model in the current investigation. The inflection point for the ALT/HDL-C ratio was 12.963 after adjusting confounding factors. It was discovered that when the ALT/HDL-C ratio was less than 12.963, every additional unit was linked to a 20.4% higher incident NAFLD. However, when the ALT/HDL-C ratio was above 12.963, a 1 unit increase was related to a 2.3% higher incident NAFLD. In Table S1, the current research demonstrated that subjects with an ALT/HDL-C ratio ≤ 12.963 had a low proportion of females and lower BMI, SBP, DBP, AST, ALP, GGT, UA, and LDL-C. However, the abovementioned factors highly correlated with NAFLD^19^. The impact of the ALT/HDL-C ratio on NAFLD was minimal when it was more than 12.963. Conversely, the risk factors associated with NAFLD were mitigated and exhibited diminished impact when the ALT/HDL-C ratio was below 12.963. At this juncture, the effect of the ALT/HDL-C ratio was significantly amplified. It has important clinical ramifications because ALT/HDL-C and NAFLD have a curvilinear correlation. When we reduced the non-obese subjects' ALT/HDL-C ratio to below 12.963 with treatments, the risk of NAFLD was reduced. Additionally, NAFLD events decreased more quickly when the ALT/HDL-C ratio decreased when it was below 12.963. The current research provides a valuable tool for clinicians to enhance consultation and improve NAFLD preventive decision-making.

The following are some of this study's advantages. (1) The sample size we used was sizable. (2) Notably, this is the first investigation to examine the correlation between ALT/HDL-C and NAFLD. (3) Strict statistical modifications were applied to reduce residual confounding variables. (4) Multiple imputations were employed to address missing data. This approach can increase statistical power while reducing bias that might result from missing covariate data. (5) we conducted several sensitivity analyses and subgroup analyses to assess the robustness of the research’s findings.

The present investigation is subject to certain limitations. First, due to its observational nature, it could not pinpoint the causal association with any degree of precision. Second, the correlation between ALT/HDL-C ratio and NAFLD in subjects with low-density lipoprotein cholesterol over 3.12 mmol/L or BMI over 25 kg/m^2^ remains unclear. This is not clear about the connection between ALT/HDL-C ratio and NAFLD in subjects with LDL-C over 3.12 mmol/L or BMI over 25 kg/m^2^. In the future, we may think about planning our trials and enlisting everyone, including patients who are lean and non-lean and who have normal and abnormal LDL-C values. Third, despite the consideration of known potential confounders, unmeasured confounders may still be present, as is typical of observational studies. Fourth, the current investigation solely assessed baseline levels of ALT, HDL-C, and other variables, without accounting for potential fluctuations in the ALT/HDL-C ratio over time. Fifth, due to its limited sensitivity, ultrasonography is not the gold standard for diagnosing NALFD. In the future, we can create our research to identify NAFLD using better techniques, including elastography.

## Conclusion

The present investigation shows a positive and non-linear association between the ALT/HDL-C ratio and NAFLD in lean Chinese individuals with a normal range of LDL-C. The relationship between the ALT/HDL-C ratio and NAFLD also has a threshold effect. The risk of developing NAFLD significantly correlates with the ALT/HDL-C ratio when it is less than 12.963. Therefore, the current study advises enhancing clinician consultation and decision-making for NAFLD prevention. Consequently, to reinforce the credibility of employing the ALT/HDL-C ratio as a conventional clinical metric for the early identification and implementation of intervention strategies in NAFLD, particularly regarding its applicability in lean cohorts, we recommend the commencement of prospective studies in real-world settings.

### Supplementary Information

Supplementary Information.

Supplementary Table S1.

## Data Availability

The authors confirm that the data supporting the findings of this study are available within the article [and/or its supplementary materials].

## Abbreviations

ALT/HDL-C ratio: Alanine aminotransferase to high-density lipoprotein cholesterol ratio
BMI: Body mass index
SBP: Systolic blood pressure
DBP: Diastolic blood pressure
ALT: Alanine aminotransferase
AST: Aspartate aminotransferase
GGT: Gamma-glutamyl transferase
ALP: Alkaline phosphatase
LDL-C: Low-density lipoprotein cholesterol
HDL-C: High-density lipoprotein cholesterol
TC: Total cholesterol
TG: Triglycerides
FPG: Fasting plasma glucose
UA: Uric acid
Cr: Creatinine
IR: Insulin resistance
GAM: Generalized additive models
T2DM: Type 2 diabetes mellitus
NAFLD: Nonalcoholic fatty liver disease
HR: Hazard ratio
SD: Standard deviations
CI: Confidence interval
