# Supplementary material for: Association between alanine aminotransferase to high-density lipoprotein cholesterol ratio and nonalcoholic fatty liver disease: a retrospective cohort study in lean Chinese individuals
Source: Sci Rep. 2024 Mar 13;14:6056. doi: 10.1038/s41598-024-56555-8 (PMC10937981; doi:10.1038/s41598-024-56555-8)
Supplement: Supplementary file 2 — Supplementary Table S1. [file 41598_2024_56555_MOESM2_ESM.docx]

**Table S1** Baseline characteristics of participants based on inflection point of ALT/HDL-C ratio

| Variable | ALT/HDL-C ratio<12.963 | ALT/HDL-C ratio≥12.963 | P-value |
| --- | --- | --- | --- |
| Participants | 6889 | 5086 |  |
| Gender |  |  | <0.001 |
| Female | 3254 (47.235%) | 2165 (42.568%) |  |
| Male | 3635 (52.765%) | 2921 (57.432%) |  |
| Age(years) | 43.167 ± 15.089 | 43.429 ± 14.739 | 0.343 |
| SBP (mmHg) | 120.205 ± 17.365 | 125.294 ± 15.887 | <0.001 |
| DBP (mmHg) | 72.285 ± 10.246 | 75.781 ± 10.219 | <0.001 |
| BMI (kg/m^2^) | 21.033 ± 2.022 | 22.365 ± 1.799 | <0.001 |
| AST (IU/L) | 19.971 ± 4.298 | 25.985 ± 8.144 | <0.001 |
| GGT(IU/L) | 22.012 ± 15.094 | 39.532 ± 39.755 | <0.001 |
| ALP(IU/L) | 67.923 ± 21.895 | 77.871 ± 23.283 | <0.001 |
| TG (mmol/L) | 1.115 ± 0.539 | 1.712 ± 1.144 | <0.001 |
| TC (mmol/L) | 4.629 ± 0.724 | 4.578 ± 0.749 | <0.001 |
| LDL-C(mmol/L) | 2.243 ± 0.470 | 2.312 ± 0.470 | <0.001 |
| FPG (mmol/L) | 5.135 ± 0.688 | 5.314 ± 0.974 | <0.001 |
| UA (umol/L) | 271.411 ± 87.712 | 322.210 ± 83.392 | <0.001 |
| Cr (umol/L) | 80.276 ± 23.371 | 87.751 ± 26.729 | <0.001 |

Values are n(%) or mean ± SD

ALT/HDL-C ratio: alanine aminotransferase to high-density lipoprotein cholesterol ratio; BMI: body mass index; SBP: systolic blood pressure; DBP: diastolic blood pressure; AST: aspartate aminotransferase; GGT: gamma-glutamyl transferase; ALP: alkaline phosphatase; TC: total cholesterol; TG: triglycerides; FPG: fasting plasma glucose, UA: uric acid; Cr: creatinine.
